# *Comment to:* Adhesive Capsulitis Secondary to COVID-19 Vaccination - A Case Series

**DOI:** 10.5704/MOJ.2403.021

**Published:** 2024-03

**Authors:** H Daungsupawong, V Wiwanitkit

**Affiliations:** 1 Consultant, Private Academic Consultant, Phonhong, Laos; 2 Research Center, Chandigarh University, Punjab, India

Dear Editor,

We would like to share ideas on the publication “Adhesive Capsulitis Secondary to COVID-19 Vaccination - A Case Series^1^.” The purpose of this study is to examine the pathogenesis, clinical manifestation, management, and outcomes of shoulder injury related to vaccine administration (SIRVA) following COVID-19 vaccination. Seven individuals with adhesive capsulitis who reported within four weeks of receiving the COVID-19 vaccine in the afflicted arm were retrospectively identified by the researchers. The study offers details on the patients' first symptoms, physical symptoms, length of time since immunisation, treatment plans, and clinical and functional results. All patients' pain levels and range of motion improved with non-surgical care, including physiotherapy and hydrodilatation.

The study's tiny sample size is a potential flaw. Since there were only seven cases, the results might not be generalisable. The results' generalisability and dependability are constrained by the short sample size. Furthermore, because the study is retrospective in nature and depends on previous medical records, recall bias and insufficient data are potential risks. A further drawback is the absence of a control group. It is difficult to ascertain the real incidence and cause of SIRVA without a control group of patients who did not experience adhesive capsulitis after receiving the COVID-19 immunisation. A control group would offer a standard against which to compare the reported cases and aid in determining if they are indeed connected to the vaccine. Additionally, the study solely considers adhesive capsulitis as a SIRVA symptom. It does not offer a thorough evaluation of any possible shoulder disorders or injuries linked to the administration of vaccines. The comprehension of the whole range of SIRVA and its clinical presentation may be constrained by this focused approach.

Before, during, or after contracting the virus, getting the COVID-19 vaccine, or at any other time, a clinical manifestation of asymptomatic COVID-19 may manifest. COVID-19 can co-occur even when no symptoms are noticeable^2^. Without the necessary laboratory testing, asymptomatic illness cannot be completely ruled out. The role of genetics is expanding. The immune system's reaction to a vaccine may differ depending on how it reacts to specific genetic features. More multicentre trials and larger sample sizes are preferred. Comprehensive epidemiological research on the relationships between disease and immunisation may be possible. comparing the prevalence of the disease in individuals who got the vaccine with those who didn't.
