# Supplementary material for: Comment to: Adhesive Capsulitis Secondary to COVID-19 Vaccination - A Case Series
Source: Malays Orthop J. 2024 Mar;18(1):155. doi: 10.5704/MOJ.2403.021 (PMC11023342; doi:10.5704/MOJ.2403.021)
Supplement: Related texts

# Author’s Reply to the Letter to the Editor

**Published:** 2024-03

**Authors:** Bryan Foong

**Affiliations:** Corresponding author Department of Orthopaedic Surgery, Tan Tock Seng Hospital, Singapore

The main purpose of our case series was to bring attention to and highlight the phenomenon of adhesive capsulitis occurring within a short period after COVID-19 vaccine administration.

We have identified within the original article and do agree that the small sample size is a limitation. Also, being a retrospective case series by design, we do appreciate that it lacks the benefit of comparison with a control group which would make the findings more robust.

We do concur with the letter to editor that a larger sample size would be beneficial, and genetics testing would offer more insight to this disease process and epidemiological research.
